# Association between cardiovascular disease and dementia

**DOI:** 10.1590/S1980-57642009DN30400008

**Published:** 2009

**Authors:** Claudia Kimie Suemoto, Renata Eloah Ferretti, Lea Tenenholz Grinberg, Kátia Cristina de Oliveira, José Marcelo Farfel, Renata Elaine Paraizo Leite, Ricardo Nitrini, Wilson Jacob Filho, Carlos Augusto Pasqualucci, Brazilian Aging Brain Study Group

**Affiliations:** 1 Department of Geriatrics, University of São Paulo, School of Medicine, São Paulo SP, Brazil.; 2 Department of Pathology, University of São Paulo, School of Medicine, São Paulo SP, Brazil.; 3 Department of Neurology, University of São Paulo, School of Medicine, São Paulo SP, Brazil.

**Keywords:** atherosclerosis, circle of Willis, carotid artery disease, cardiomyopathy, dementia

## Abstract

Longitudinal studies have shown association between cardiovascular risk factors and dementia. However, these studies are not capable of detecting asymptomatic cardiovascular alterations and thus may provide erroneous estimates of association. Autopsy studies could be more useful in elucidating these questions. The present clinicopathological study sought to examine the relationship between dementia, cardiovascular risk factors and disease.

**Methods:**

603 subjects, who underwent autopsy, were classified regarding the presence of dementia, according to *post mortem* cognitive classification. Demographics, cardiovascular risk factors, and anatomically-proven cardiovascular disease (myocardial hypertrophy, cerebral and carotid atherosclerosis) were compared among cognitively normal persons and individuals with dementia.

**Results:**

Cognitive deficit was associated with advanced age, stroke, physical inactivity and low body mass index (p< 0.05). Circle of Willis atherosclerosis was greater in patients with dementia than in controls on univariate analysis (p=0.01). However, this association lost significance when adjusted by age and gender (p=0.61). Heart failure and anatomopathological cardiac parameters were more severe in the control group than in demented individuals (p< 0.05). Carotid artery atherosclerosis and intima-media thickness were similar in both groups.

**Conclusion:**

Advanced age, stroke, physical inactivity and low body mass index were linked to dementia. Circle of Willis atherosclerosis was associated with dementia only when age was not considered. Our results suggest that cerebral artery atherosclerosis was not directly associated with clinical expression of dementia.

## Abstract (pt)

Estudos longitudinais tem mostrado associação entre fatores de risco cardiovascular e demência. Entretanto, estes estudos não são capazes de detectar alterações cardiovasculares assintomáticas e podem, assim, fornecer estimativas de associação errôneas. Estudos de autópsia podem ser mais úteis em elucidar estas questões. O presente estudo clinicopatológico busca examinar a relação entre demência, fatores e doença cardiovascular.

**Métodos:**

603 sujeitos submetidos à autópsia foram classificados quanto à presença de demência, usando uma classificação cognitiva *post-mortem*. Dados demográficos, fatores de risco cardiovascular e doença cardiovascular comprovada anatomicamente (hipertrofia miocárdica, aterosclerose cerebral e carotídea) foram comparados entre indivíduos cognitivamente normais e com demência.

**Resultados:**

Déficit cognitivo esteve associado à idade avançada, acidente vascular cerebral, sedentarismo e baixo índice de massa corpórea (p< 0,05). Aterosclerose do polígono de Willis foi maior em pacientes com demência do que em controles na análise univariada (p=0,01). Insuficiência cardíaca e parâmetros anatomopatológicos cardíacos foram mais graves entre o grupo controle que entre os indivíduos dementados (p< 0,05). Aterosclerose de artérias carótidas e espessura íntima-média foram similares entre os grupos.

**Conclusão:**

Idade avançada, acidente vascular cerebral, sedentarismo e baixo índice de massa corpórea estiveram relacionados à demência. Aterosclerose de polígono de Willis esteve associada à demência, somente quando a idade não foi considerada. Nossos resultados sugerem que a aterosclerose de artérias cerebrais não está diretamente relacionada com a expressão clínica de demência.

Evidence derived from epidemiologic studies links cardiovascular risk factors (CVRF), Alzheimer’s disease, and vascular dementia.^1-4^ Hypertension, hypercholesterolemia, diabetes mellitus, obesity, metabolic syndrome, smoking, hyperhomocysteinemia, and apoliproteinemia Eε4 are risk factors linked to the development of dementia^5^. The presence of cardiovascular diseases such as atrial fibrillation, peripheral artery disease, carotid artery atherosclerosis, coronary artery disease, stroke and heart failure are also associated with a greater prevalence of dementia, including AD.^6^

Although there is much evidence for the coexistence of CVRF and dementia, particularly AD, it is unclear whether this relationship is causal or coincidental. Recent reports suggest that atherosclerosis of the circle of Willis (CW) is more severe in individuals with AD than in control subjects.^7,8^ The degree of stenosis caused by atherosclerosis in the CW was found to correlate with neuropathological lesions characteristic of AD, leading to the hypothesis of a causal relationship between atherosclerosis and neurodegenerative disease.^9^ These results were obtained by analyzing the brains of old subjects with advanced dementia.

The present study investigated the relationship between CVRF, pathologically-proven cardiovascular disease (myocardial hypertrophy, carotid artery and CW atherosclerosis) and dementia in an extensive sample group that included persons with mild to severe dementia.

## Methods

### Participants, demographics and cognitive evaluation

The study was performed drawing from the Human Brain Bank of the Brazilian Aging Brain Study Group (BABSG)^10^ from April of 2005 to December of 2008. All deceased individuals over 50 years were selected, excluding cases with: death from primary cerebral disease, potential cerebral ischemic lesions due to hypoperfusion, cerebrospinal fluid acidosis due to terminal condition, and lack of a caregiver to provide adequate information about the deceased. Brains with acute stroke were not provided to the BABSG because of the need to examine these organs to produce an autopsy report. The present study was approved by the ethics and research committee of the University of São Paulo Medical School, and all the informants signed an informed consent form.

Information about demographics and CVRF were collected through structured interviews from an informant who spent time with the deceased at least once a week. The cause of death was obtained from the autopsy report.

Cognitive status was assessed post-mortem, using information provided during the clinical interview. The Clinical Dementia Rating (CDR)^11^ questionnaire was used to evaluate cognitive functions. The participants were clinically classified for the presence of dementia and its severity (without dementia=CDR 0; mild dementia=CDR 1; moderate dementia=CDR 2; and severe dementia=CDR 3). Individuals with questionable dementia (CDR=0.5) were excluded. To further support the diagnosis of dementia, the IQCODE (Informant Questionnaire on Cognitive Decline in the Elderly) scale was used. A cut-off value of 3.4 was adopted to discriminate between individuals with and without cognitive impairment.^12^ Individuals with CDR ≥1 and IQCODE ≥3.4 were considered demented. The control group consisted of subjects with CDR=0 and IQCODE < 3.4.

### Cardiovascular evaluation

**Figure 1:**
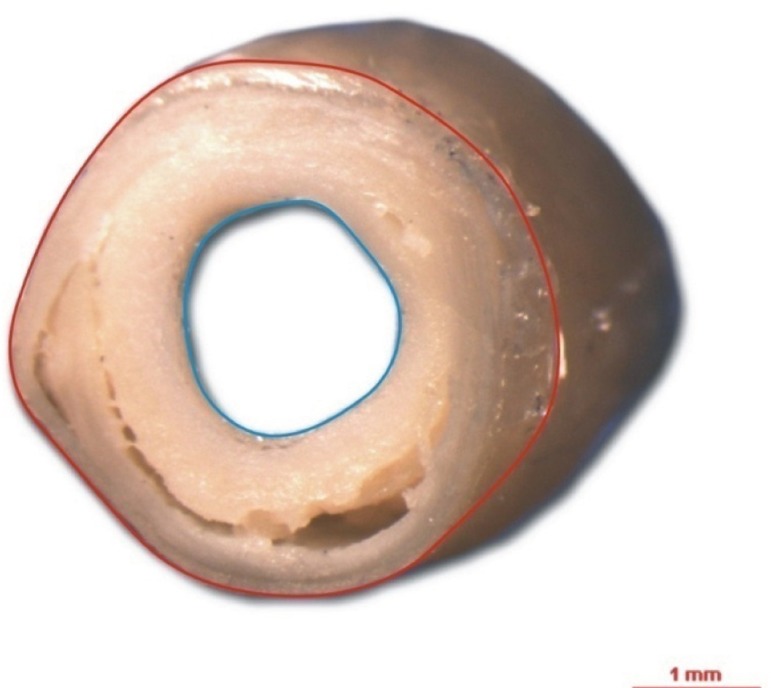
Cross-sectional image of basilar artery from the circle of Willis. The red tracer is placed on the outer circumference of the artery and the blue inner tracer on the circumference of the lumen. Calibration bar=1 mm.

The CW was conserved in 70% ethyl alcohol until the injection of agar in the vessel lumen was performed in order to avoid collapse of the artery. It was subsequently fixed in 10% paraformaldehyde. The anterior cerebral arteries, anterior communicating artery, middle cerebral arteries, internal carotid arteries, posterior communicating arteries, posterior cerebral arteries and basilar artery were dissected and cut into 3 mm-long slices. The slice section with the greatest obstruction was photographed with a stereomicroscope (Nikon^®^ SMZ 1000). For each slice section, the lumenal and outer areas were measured using the ImageJ^®13^ image processing program (Figure 1). The percentage of arterial obstruction was calculated subtracting the lumenal area from the outer area of the vessel and dividing the difference by the outer area and multiplying the quotient by 100.

Carotid arteries were also conserved in 70% ethyl alcohol until the injection of agar and fixation with 10% paraformaldehyde. They were subsequently cut into 5 mm lengths. Four segments of each side of the carotid system were considered:

(1) section with the greatest obstruction of the common carotid artery;
(2) section of the common carotid artery at 1 cm below bifurcation between this artery and internal carotid artery;
(3) section of the internal carotid artery 1 cm after bifurcation; and
(4) section of the internal carotid artery with the greatest obstruction.

These slices were embedded in paraffin and cut into 8 µm-thick sections. Each of these regions was stained using Verhoeff’s stain and photographed with a stereomicroscope. The luminal and intimal areas were calculated using the image analyzer. The intima area was delineated by the internal elastic lamina up to the endothelial surface. The percentage of carotid stenosis was calculated by subtracting the luminal area from the intima area, dividing the difference by the intima area, and multiplying by 100. The following measurements were used to calculate the intima-media thickness (IMT):

(a) the intima-media area, defined as the area internal to the external elastic lamina up to the lumen;
(b) the media perimeter, delineated by the external elastic lamina (Figure 2).
  **Figure 2:**
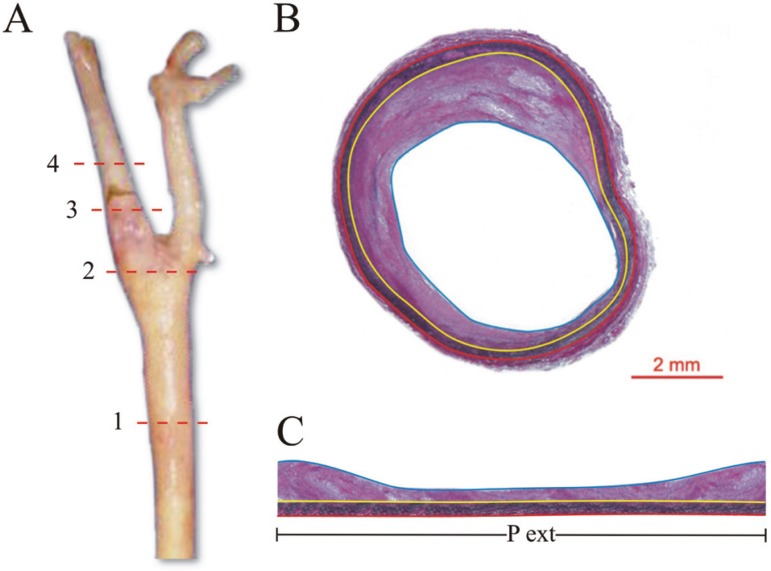
(A) Right carotid artery and the site of 4 cross-sections: (1) the greatest common carotid artery stenosis; (2) 1 cm below carotid artery bifurcation; (3) 1cm above carotid artery bifurcation; (4) the greatest internal carotid artery stenosis; (B) Histological representation of common carotid artery using Verhoeff’s stain. Red tracer: external elastic lamina; yellow tracer: internal elastic lamina; blue tracer: lumen. (C) Flat section of common carotid artery (Figure 2B). For the calculation of intima media thickness, the intima-media area was divided by the external perimeter (area between red and blue tracer), delimited by external elastic lamina (red). Pext=perimeter of external elastic lamina. Calibration bar=2 mm.

To calculate IMT, the luminal area was subtracted from the intima-media area, dividing the difference by the media perimeter.

The hearts were fixed in 10% paraformaldehyde and weighed on electronic scales. The thickness of the free wall of the left ventricle was measured 1 cm below the aortic valve. Each case was classified as to the presence of myocardial hypertrophy of the left ventricle, taking into consideration heart weight, thickness of left ventricle, age and gender of the individual.

### Statistical analysis

Frequency distribution of demographics and CVRF were analysed using the descriptive statistics mode of the SPSS program (version 14.0). For categorical variables, the Chi-squared test was used to compare individuals with and without dementia. Fisher’s exact test was selected, when necessary. For continuous variables, a nonparametric test (Mann-Whitney test) was used to compare the two groups. The associations that were significant on univariate analysis were evaluated in a logistic regression model. The association between cardiovascular disease (cardiac variables and CW atherosclerosis) and dementia were adjusted for age and sex also using the logistic regression model. The level of significance of all tests was set at 5%.

## Results

**Figure 3:**
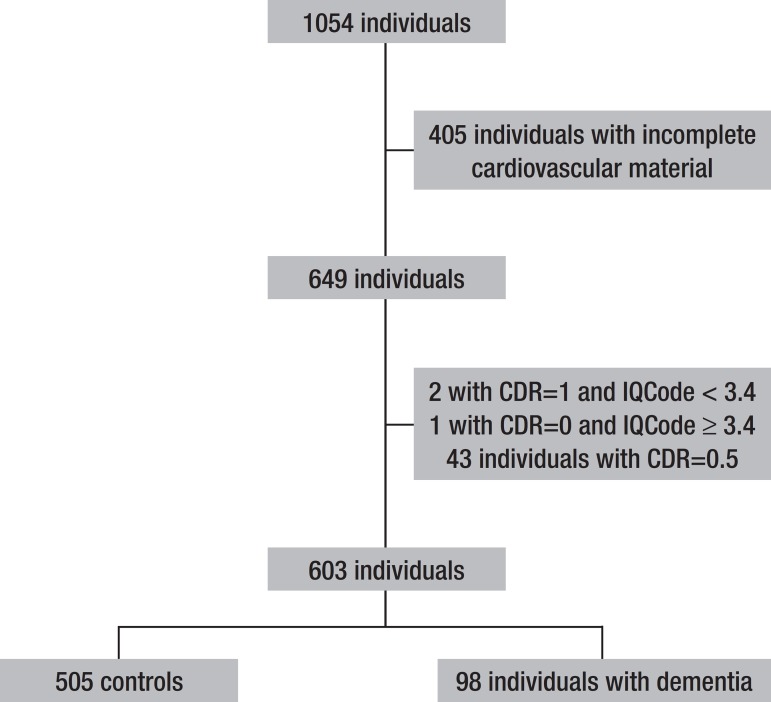
Flow diagram of the participants of the study.

Figure 3 shows the flow diagram of the analyzed cases. The 603 participants of the study were similar to the 451 individuals that were excluded from the study in terms of the distribution of age, sex, education level and proportion of dementia. Based on the CDR scale, individuals with dementia (n=98; 16.3%) were classified as mild dementia (n=33; 33.7% of demented individuals), moderate (n=21; 21.4%) or advanced dementia (n=44; 44.9%).

Table 1 shows demographics and CVRF of controls, and subjects with dementia. On univariate analysis, individuals with dementia were older (p< 0.001) and had less years of education (p< 0.001) than the control group. There were fewer men (p< 0.001) and cardiovascular cause of death (p< 0.001) among demented compared to control individuals. Heart failure was more common among controls (p=0.03). The dementia group had more stroke and physical inactivity (p=0.03) and less smoking (p< 0.001) and drinking (p=0.001) than the control group. Body mass index (BMI) was lower in demented individuals than in controls (p< 0.001). After logistic regression analysis only age, heart failure, stroke, physical inactivity and low BMI remained associated with dementia.

**Table 1:** Demographics and cardiovascular risk factors among individuals with dementia and without dementia (controls). BABSG, 2005-2008.

	Controls	Dementia	p
Age at death, years, mean (SD)	69.2 (11.4)	79.4 (9.6)	<0.001
Education, years, mean (n)	4.5 (3.7)	3.1 (3.1)	<0.001
Male, n (%)	276 (54.7)	33 (33.7)	<0.001
Caucasian, n (%)	336 (67.2)	70 (71.4)	0.69
Married (marital status), n (%)	232 (46.1)	28 (28.6)	0.001
Cardiovascular cause of death, n (%)	331 (65.5)	52 (53.1)	<0.001
Social class C, n (%)	241 (47.7)	44 (44.9)	0.16
Hypertension, n (%)	331 (65.5)	55 (56.1)	0.08
*Diabetes mellitus, *n (%)	139 (27.5)	28 (28.6)	0.83
Coronary arterial disease, n (%)	142 (28.1)	18 (18.4)	0.05
Heart failure, n (%)	104 (20.6)	11 (11.2)	0.03
Arrhythmia, n (%)	49 (9.7)	6 (6.1)	0.26
Dyslipidemia, n (%)	52 (10.3)	5 (5.1)	0.11
Stroke, n (%)	43 (8.5)	24 (24.5)	<0.001
Physical inactivity, n (%)	291 (57.6)	83 (84.7)	<0.001
Smoking, n (%)	138 (27.3)	13 (13.3)	<0.001
Drinking, n (%)	133 (26.3)	15 (15.3)	0.001
BMI, kg/m^2^, mean (SD)	23.6 (4.5)	21.4 (3.9)	<0.001
Total, n	603	98	

SD, standard deviation; BMI, body mass index.

Regarding anatomically-proven cardiovascular disease, dementia subjects had the greatest percentage of CW arterial obstruction (p=0.02) as well as the greatest number of stenosis per case (p=0.01) compared with controls (Table 2). The right posterior cerebral artery, right and left middle cerebral arteries and right and left anterior cerebral arteries had more obstruction in demented patients than in controls (p< 0.05). However, this association lost significance when logistic regression was applied (Table 3).

**Table 2:** Odds ratio for dementia, regarding demographic profile, cardiovascular risk factors and dementia. BABSG, 2005-2008.

Variable	OR (95% CI)*	p
Age	1.07 (1.04-1.10)	< 0.001
Male gender	0.90 (0.49-1.63)	0.72
Not married	1.11 (0.60-2.06)	0.75
Years of education	0.95 (0.87-1.03)	0.21
Cardiovascular cause of death	0.95 (0.55-1.64)	0.86
Coronary artery disease	0.98 (0.52-1.86)	0.95
Heart failure	0.45 (0.21-0.97)	0.04
Stroke	3.32 (1.65-6.69)	0.001
Physical inactivity	3.80 (2.02-7.14)	<0.001
Smoking	0.86 (0.60-1.24)	0.43
Drinking	0.72 (0.47-1.12)	0.15
Body mass index	0.92(.86-0.98)	0.01

* logistic regression model; OR, odds ratio; 95% CI, 95% confidence interval.

**Table 3:** Comparison of circle of Willis atherosclerosis between controls and dementia group. Odds ratio for dementia, adjusted for age and gender. BABSG, 2005-2008

Circle of Willis	Controls	Dementia	OR (95% CI)	p
Arterial obstruction (%), mean (SD)	16.8 (16.9)	21.3 (18.3)	1.00 (0.99-1.02)	0.61
Number of arteries with atheroma/CW, mean (SD)	3.31 (2.9)	4.13 (3.0)	1.01 (0.94-1.10)	0.74
Number of arteries with obstruction>50%/CW, mean (SD)	2.01 (2.44)	2.60 (2.62)	1.02 (0.93-1.12)	0.66

SD, standard deviation.

The groups were similar in relation to carotid artery atherosclerosis and IMT. The cardiac weight was greater in controls than in individuals with dementia (397.1±131.4g vs 321.8±93.5g, p< 0.001). The left ventricle wall thickness was greater in the control group than in the dementia group (12.7±2.6mm vs 11.8±2.4mm, p=0.002). Furthermore, the controls had more myocardial hypertrophy than the demented individuals (45.7% vs 31.6%, p=0.01). After adjustment for age and gender, low cardiac weight (p< 0.001) and thin left ventricle wall (p=0.01) remained associated with dementia.

## Discussion

Our results showed an association between CW artery atherosclerosis and dementia. CW atherosclerotic disease was both most severe and extensive in individuals with dementia than in controls. However, these associations lost significance after adjustment for age and gender. In our sample, demented individuals were older than control subjects. As advanced age is associated both with increased risk of dementia^14^ and with higher burden of atherosclerotic lesions,^15^ age was a confounding factor in our study. Similarly, a previous study showed that CW cerebral atherosclerosis was not directly associated with AD neuropathological lesions.^16^ Amyloid plaque load and neurofibrillary tangle count were associated only with age after linear regression model adjustment.

We found no association between carotid stenosis and dementia. This corroborates the results of previous studies which also found no association between carotid stenosis and dementia.^17,18^ However, Johnson et al. showed cognitive deficit to be associated only with critical lesions, greater than 75%, in a cohort of more than 4,000 patients.^19^ A transverse study conducted in Rotterdam described a strong association between carotid artery disease and dementia, showing that common carotid artery plaques and IMT were associated with a greater risk of dementia, including AD and VaD.^20^ However, a follow up of the cited study involving 6,647 participants showed no association between carotid artery plaques and dementia. The authors stated that the link found between atherosclerosis and dementia was stronger at the beginning of the study, and lessened with time, probably due to the impact of atherosclerosis on mortality.^21^ Although the IMT of these arteries is linked with a higher risk of cerebral lesions,^22^ we found no association with dementia in our study. Previous investigations have shown increased carotid artery IMT to be associated with a greater risk of AD, but only when comparing IMT between extreme groups (those of the first and last quartiles).^17,18,21^ All of the studies cited employed ultrasonography to determine IMT and arterial stenosis. However, quantitative measures using autopsy material are more accurate and therefore the disparities observed could have been due to the different methods employed.^23^

Controls had higher heart weight and increased thickness of the left ventricle compared to the dementia group. Other previous clinicopathological studies failed to find association between cardiac parameters and dementia.^24-26^

Most of these studies examined subjects with advanced dementia. The malnutrition and consequent low cardiac weight found in such individuals had been proposed as an explanation for these findings. Corroborating this idea, in our study, the dementia group showed lower body mass index than the control group and this fact could explain the association between low cardiac weight and thinner left ventricle wall in demented individuals.

Regarding demographics and CVRF, this study showed an association between increasing age and dementia. Advanced age is the risk factor most consistently associated with dementia. In Brazil, as well as in other countries in the world, an exponential increase in the prevalence of dementia with aging has already been described.^27,28^ The presence of heart failure was more common among the control group. In contrast to our results, previous studies showed that heart failure was a risk factor for dementia.^29^

Reporting of stroke by informants was more common in individuals with dementia than in controls. The importance of cerebral infarcts in the clinical expression of dementia has been previously described, indicating that the presence of ischemic lesions results in cognitive disturbance with fewer neuropathological alterations associated with AD.^30,31,33^ Physical inactivity was linked to dementia in our study. Similarly, sedentary life style had been considered a risk for dementia in previous studies.^34^

The advantages of our study include the breadth of the clinical information and the heterogeneity of the sample, including subjects with mild to severe dementia. The anatomically-proven cardiovascular analysis is a direct measure, which made the evaluation less influenced by the subjectivity of the investigator. Moreover, quantitative measurement of arterial stenosis, although laborious, has not previously been employed on a large scale.

The drawbacks of our study stem from the cross sectional design of autopsy studies, which can hamper the establishment of causal relationships. Moreover, it is important to note that the cognitive evaluation was based on *post mortem* information provided by caregivers, while the CDR was originally developed for use in patients and their guardians. Nevertheless, this approach has been validated^35^ and applied in previous autopsy studies.^36,37^ The use of IQCODE in *post mortem* evaluation has previously been described.^38^ The combination of these two scales should improve the accuracy of the dementia diagnosis in our sample. Another disadvantage is that neuropathology was not performed in this study and therefore it was not possible to investigate the interaction between cardiovascular data and cerebral lesions. Although an association between apolipoprotein Eε4, dementia and CVRF is known,^16^ the apolipoprotein E4 profile was not available in our sample.

In spite of the cited limitations, the information obtained confirms previous evidence linking some CVRFs, particularly stroke and physical inactivity, to higher risk of dementia. Although the underlying reason for this association remains unknown, preventive measures aimed at controlling risk factors may be an effective means of delaying the development of dementia. Circle of Willis atherosclerosis, which was previously linked to increased dementia risk, was not directly associated with cognitive deficit in our study, which instead showed the importance of the interaction between age and the development of dementia and atherosclerosis.
